# Supplementary material for: Quality versus quantity of training datasets for artificial intelligence–based whole liver segmentation
Source: medRxiv. 2026 Feb 18:2026.02.17.26346486. Preprint. [Version 1] doi: 10.64898/2026.02.17.26346486 (PMC12934849; doi:10.64898/2026.02.17.26346486)
Supplement: Supplement 1 [file media-1.pptx]

## Slide 1
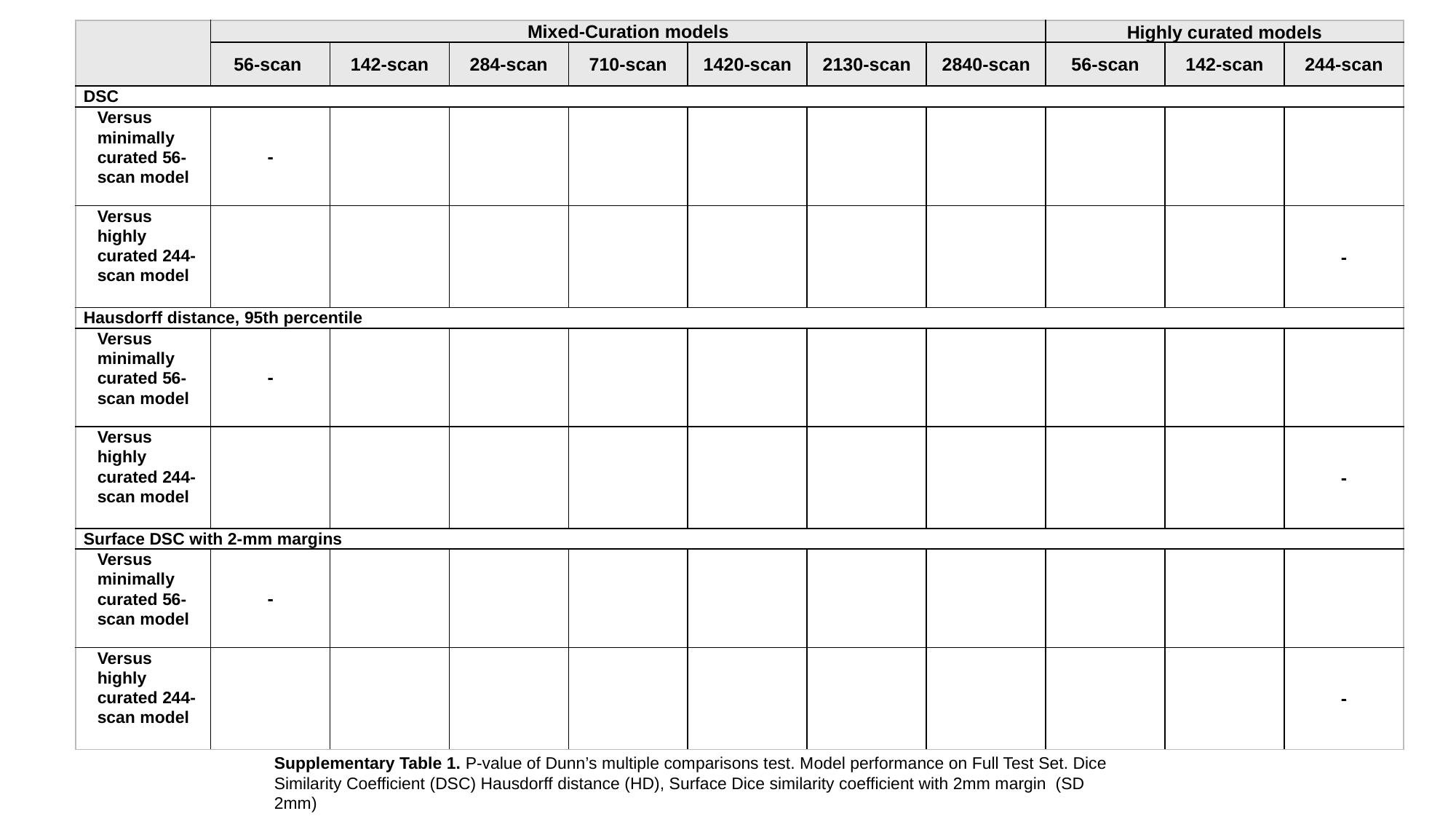

Supplementary Table 1. P-value of Dunn’s multiple comparisons test. Model performance on Full Test Set. Dice Similarity Coefficient (DSC) Hausdorff distance (HD), Surface Dice similarity coefficient with 2mm margin (SD 2mm)

## Slide 2
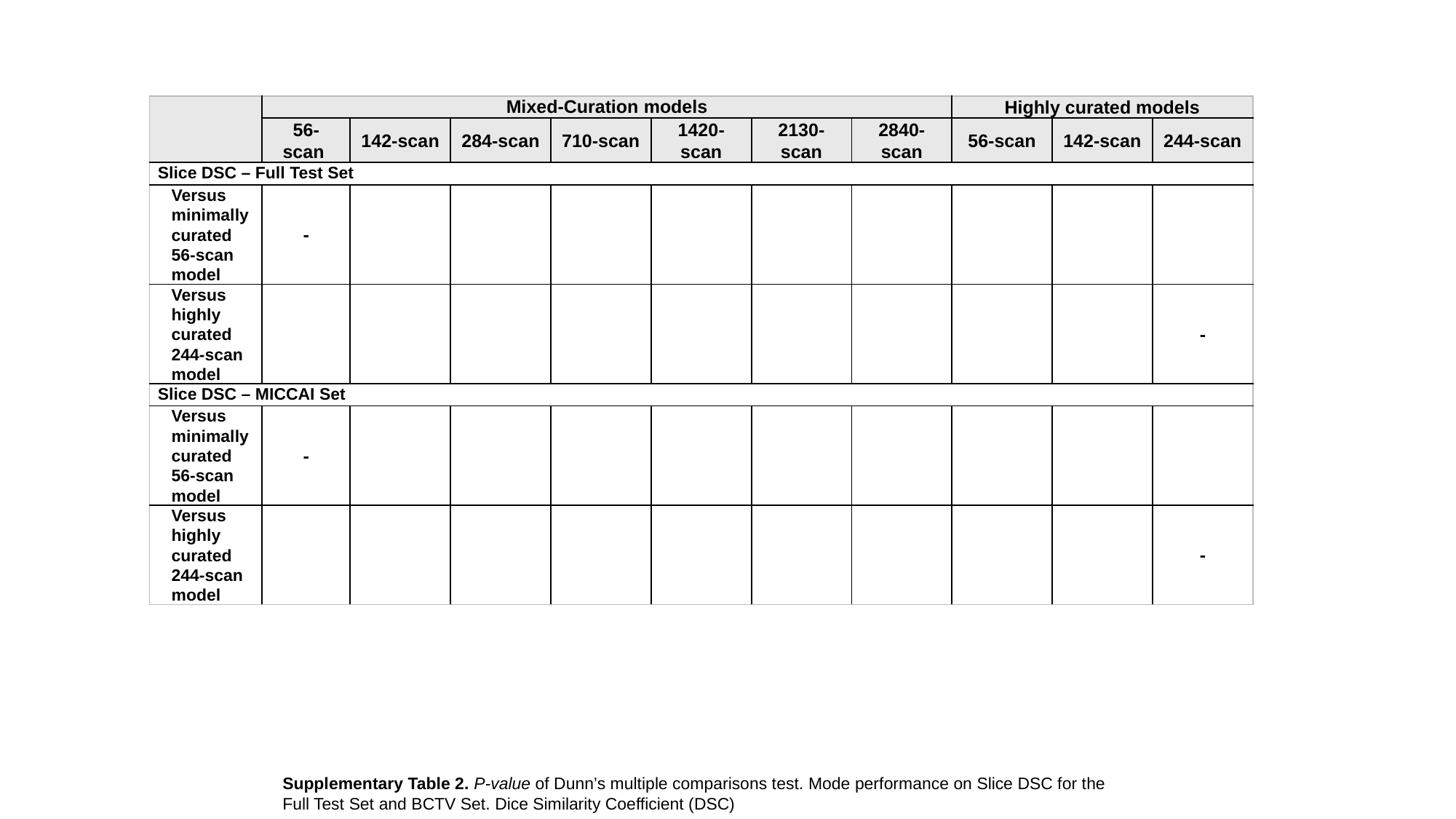

Supplementary Table 2. P-value of Dunn’s multiple comparisons test. Mode performance on Slice DSC for the Full Test Set and BCTV Set. Dice Similarity Coefficient (DSC)

## Slide 3
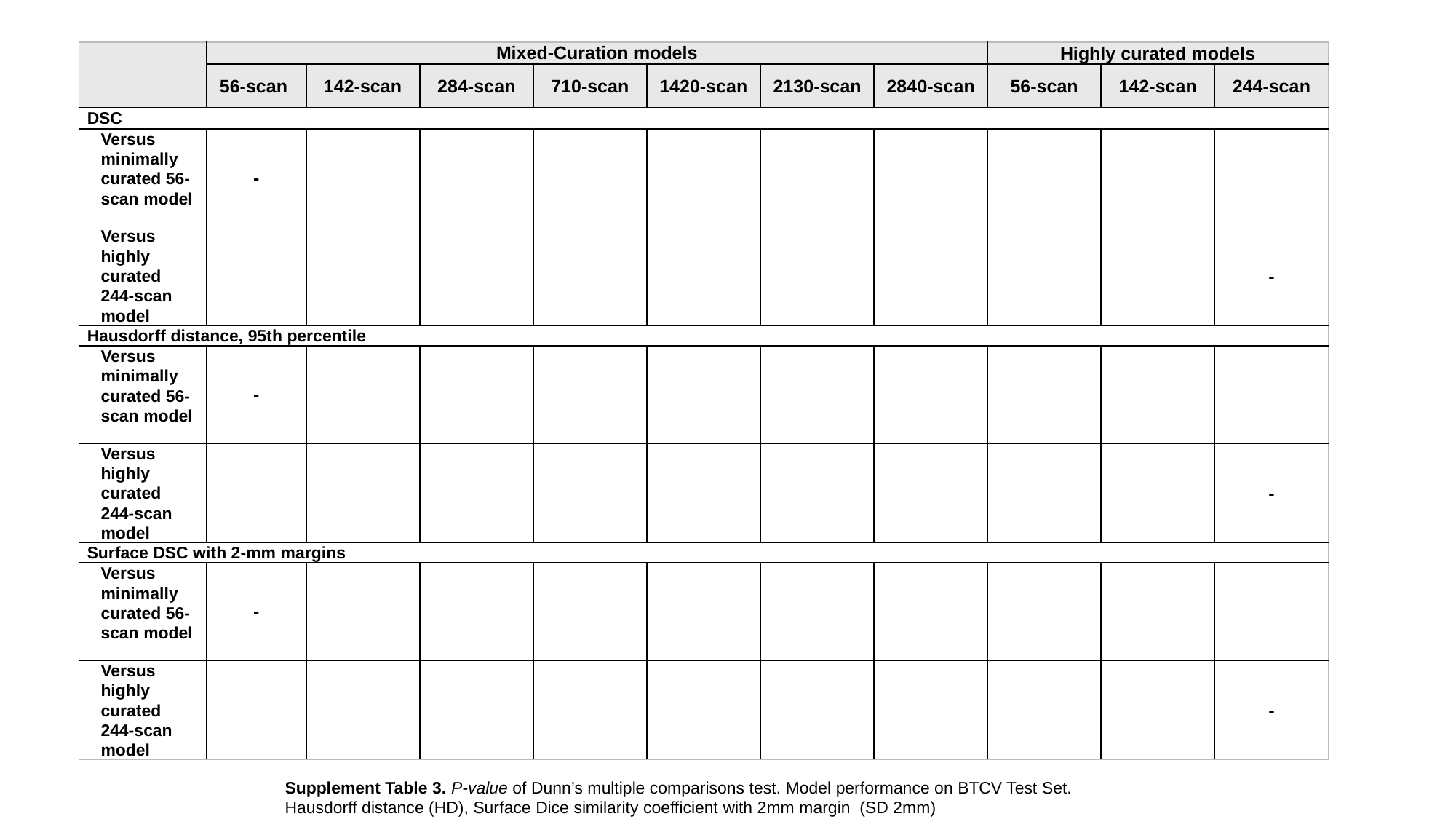

Supplement Table 3. P-value of Dunn’s multiple comparisons test. Model performance on BTCV Test Set. Hausdorff distance (HD), Surface Dice similarity coefficient with 2mm margin (SD 2mm)
